# Epigenetic alterations following early postnatal stress: a review on novel aetiological mechanisms of common psychiatric disorders

**DOI:** 10.1186/s13148-015-0156-3

**Published:** 2015-11-14

**Authors:** Magdalene C. Jawahar, Chris Murgatroyd, Emma L. Harrison, Bernhard T. Baune

**Affiliations:** Discipline of Psychiatry, School of Medicine, University of Adelaide, Adelaide, SA 5005 Australia; School of HealthCare Science, Manchester Metropolitan University, Manchester, UK; School of Medicine and Dentistry, James Cook University, Townsville City, Australia

**Keywords:** Early life stress, Maternal separation, Epigenetics, DNA methylation, Stress-responsive genes, Histone acetylation, Psychopathology

## Abstract

Stressor exposure during early life has the potential to increase an individual’s susceptibility to a number of neuropsychiatric conditions such as mood and anxiety disorders and schizophrenia in adulthood. This occurs in part due to the dysfunctional stress axis that persists following early adversity impairing stress responsivity across life. The mechanisms underlying the prolonged nature of this vulnerability remain to be established. Alterations in the epigenetic signature of genes involved in stress responsivity may represent one of the neurobiological mechanisms. The overall aim of this review is to provide current evidence demonstrating changes in the epigenetic signature of candidate gene(s) in response to early environmental adversity. More specifically, this review analyses the epigenetic signatures of postnatal adversity such as childhood abuse or maltreatment and later-life psychopathology in human and animal models of early life stress. The results of this review shows that focus to date has been on genes involved in the regulation of hypothalamic-pituitary-adrenal (HPA) axis and its correlation to subsequent neurobiology, for example, the role of glucocorticoid receptor gene. However, epigenetic changes in other candidate genes such as brain-derived neurotrophic factor (*BDNF*) and serotonin transporter are also implicated in early life stress (ELS) and susceptibility to adult psychiatric disorders. DNA methylation is the predominantly studied epigenetic mark followed by histone modifications specifically acetylation and methylation. Further, these epigenetic changes are cell/tissue-specific in regulating expression of genes, providing potential biomarkers for understanding the trajectory of early stress-induced susceptibility to adult psychiatric disorders.

## Background

Early life stress (ELS) encompasses childhood abuse, neglect, poverty and parental illness, alongside a multitude of other stressors. Some forms of ELS affect 30–40 % of the Western population and have been implicated in approximately half of all childhood and a third of adulthood psychiatric disorders [1, 2]. Exposure to ELS results in enhanced susceptibility to neuropsychiatric disorders such as major depressive disorder, generalised anxiety disorder, schizophrenia and autism spectrum disorders [1, 3, 4]. Chronic health conditions such as obesity, type two diabetes mellitus, respiratory disorders and cardiovascular diseases are also increased in individuals with a history of ELS [5, 6].

The impact of early adversity on the susceptibility to psychiatric disorders in later life is influenced by a number of factors. Environmental factors include nature of stressors [7], time of exposure in development [8, 9] and severity and cumulative exposure effects [10–12]. Biological factors include gender, age of assessment [13] and predisposing genetic polymorphisms in genes associated with mood regulation, stress response and inflammatory processes. These include genes such as serotonin transporter (*5-HTT*), brain-derived neurotrophic factor (*BDNF*) and FK506 binding protein (*FKBP5*) [14–16]. A dysfunctional hypothalamic-pituitary-adrenal (HPA) stress axis and impaired immune responses such as increased cytokines have also been implicated in the increased vulnerability to ELS [17, 18]. In spite of the increasing knowledge, the molecular mechanisms underlying ELS-mediated long-term vulnerability to later-life stressors are unclear.

Gene-environment interactions, such as those occurring when exposed to ELS, often encompass epigenetic changes. Epigenetics processes occur at the level above the genome, which includes DNA methylation, posttranslational histone modifications and gene regulation by micro-RNA (miRNA). Collectively, these epigenetic changes can stably mark the genome in response to environment, potentially altering gene expression across lifespan [19, 20] and across generations [21]. As such, epigenetic alterations may represent one of the key mechanisms underlying the long-lasting nature of ELS-induced changes in neurobiology, behaviour and disease susceptibility [22, 23].

We first present a brief overview of the stress response pathways followed by a detailed review of evidence demonstrating epigenetic alterations following ELS in animal models and humans. Changes in the epigenetic signature of candidate genes and alterations in genome-wide methylation will be reported. Finally, we aim to understand how these changes mediate long-term effects such as their role in risk to developing psychiatric disorders in adulthood.

### The central role of hypothalamus-pituitary-adrenal system

**Fig. 1:**
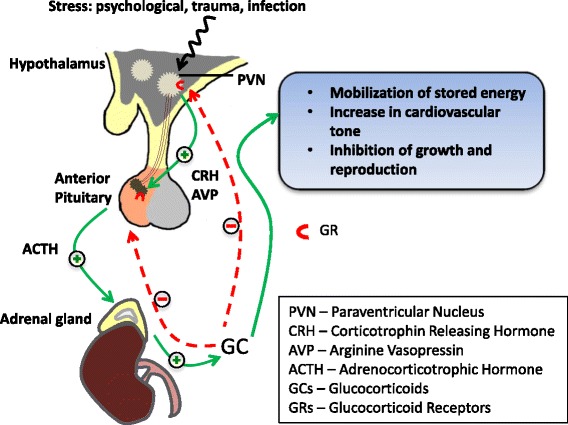
The hypothalamic-pituitary-adrenal (HPA) axis and its response to stress stimuli: the signalling events (*green*, *solid lines*) in the HPA axis in response to stress stimuli and how glucocorticoids (GCs) produced by the adrenal gland can have a negative feedback role in maintaining GC levels in the blood. The negative feedback in the hypothalamus and pituitary (*red*, *dotted lines*) are both mostly regulated by glucocorticoid receptors (GRs), and dysfunctional negative feedback system is often seen associated with chronic exposure to stress stimuli

Adversities during early postnatal life are able to shape the experience-dependent maturation of stress-regulating pathways, such as the HPA system. This can lead to persistent alterations in stress responsivity during adulthood—a phenomenon often referred to as “early-life programming”. Tight regulation of the HPA axis is therefore core to the long-term control of systems governing stress responsivity. The HPA axis involves the release, following a stressor, of the neuropeptide corticotrophin-releasing hormone (CRH) and arginine vasopressin (AVP) from the paraventricular nucleus (PVN) of the hypothalamus. These bind to their specific receptors (the CRHR1 and V1b receptors) in the anterior pituitary that stimulate the release of adrenocorticotrophic hormone (ACTH) which stimulates the adrenal cortex to release glucocorticoid (GC) hormones, cortisol in human and corticosterone in rodents. These GCs in turn mobilise glucose from energy stores and increase cardiovascular tone, among further widespread effects. Feedback loops, primarily mediated through glucocorticoid receptors (GRs) in the PVN and pituitary, regulate responsiveness of the HPA axis ensuring a return to a homeostatic balance when it is no longer challenged (Fig. 1).

A loss of this negative feedback control, particularly following periods of chronic stress, may influence the development of affective disorders. Indeed, dysregulated HPA activity is one of the most commonly observed neuroendocrine symptoms in major depressive disorder (for review see [24]). Childhood stress has also been shown to be a strong predictor of impaired inhibitory feedback regulation of the HPA axis with evidence linking to a role of CRH and/or AVP systems. For example, postmortem brain tissue of depressed individuals revealed elevated CRH and AVP in the hypothalamus [25, 26]. Studies in rodent models further support the concept that exposure to a chronic stressor can lead to long-term changes in HPA regulation and behaviour stemming from changes in neuropeptide regulation (for review see [27]).

### The role of candidate genes outside of the HPA axis

Candidate genes such as the serotonin transporter *SLC6A4* and *BDNF* have been highly implicated in stress response and in increased risk for psychiatric disorders [28–32]. BDNF is the most prevalent growth factor in the central nervous system (CNS) and important in neuronal development and plasticity [33]. Serotonin transporter is involved in the reuptake of serotonin from the brain synapses regulating serotonin signalling and is the target for many antidepressants [34]. *SLC6A4* or 5*-HTT* carries a genetic polymorphism in the promoter region resulting in a short “s” and a long “l” allele version of the promoter [35]. The “s” allele is associated with poor transcriptional efficiency of *SLC6A4* compared to “l” allele [35]. The BDNF gene carries a Val66Met polymorphism which impacts an activity-dependent expression of BDNF and the intracellular trafficking [36]. In combination with exposure to ELS events, both *SLC6A4* and *BDNF* polymorphisms have been attributed to increased risk for depression in later life [28, 30, 37]. Further, steroid hormone estrogen and its receptors have been shown to influence brain function and psychiatric disorders (for review see [38]). Animal models analysing maternal care in rats identified estrogen receptor α expression was altered with the type of maternal care, and this was passed across generations [39]. A detailed analysis of the role of these and other candidate genes implicated in ELS and later-life psychopathology is reviewed in the following sections.

## ELS-induced epigenetic modifications in animal models

A variety of animal models are currently being used to model ELS paralleling childhood adversity in humans (Table 1). Each paradigm facilitates investigation into ELS-induced alterations in the developing animal and centres on the importance of mother for normal nervous, immune and endocrine system development [40–42]. A majority of the literature on animal models discussed in this review will therefore be on variations in maternal care.

**Table 1:** Commonly used models of early adversity in animal studies

Paradigm	Description	Rationale	References
Handling	Daily separation of pups as a litter from mother for 15 min from PND1–14 (up to PND21)	Allows comparison of mild vs severe stress-induced effects during the early postnatal period	[94]
Early weaning	Weaning of pups between PND14 and PND21	Enables assessment of prolonged stress after the early postnatal period	[42, 95]
Maternal separation	Daily separation of pups individually or as a litter from mother for 3 h (up to 6 h) from PND1–14 (up to PND21)	Ethnologically relevant as it models repeated episodes of mild stress rather than 1 episode of severe stress during the early postnatal period	[96]
Maternal deprivation	Single episode of separation of pups as a litter from mother for 24 h, usually on PND3 or 9	Allows determination of stress-induced effects at specific developmental time windows within the early postnatal period	[41, 47, 95]
Low vs high LG-ABN	Pups raised by biological mothers who are characterised as either low or high care dams on the basis of the level of licking grooming arched-back nursing they perform	Enables comparison of the effects of variations in maternal care on development rather than stress exposure	[39, 97]

*h* hour, *LG-ABN* licking grooming arched-back nursing, *PND* postnatal day

### ELS-induced epigenetic changes in HPA axis genes

Given the central role of the HPA axis in stress responsivity and adaptation to ELS, the genes involved in regulating this system have been of focus in ELS-induced epigenetic studies (see Table 2 for summary of studies).

**Table 2:** Early stress-induced epigenetic changes in stress-regulatory genes in animal studies

Candidate gene	Objective	Model/tissue type	Early stress/assessment age	Epigenetic and expression changes	Interpretation	References
GR promoter	Determine the effect of maternal care on exon 1_7_ GR promoter methylation and histone H3K9 acetylation	Long-Evans rats	Maternal care variations: high vs low LG-ABN Assessed: PND6, 21 and 90	↓ methylation 5′ CpG of NGFI-A response element in high vs low LG-ABN offspring (↔3′ CpG) ↑ histone H3K9 acetylation in high vs low LG-ABN offspring ↑ NGFI-A binding to exon 1_7_ GR promoter in high vs low LG-ABN offspring ↑ GR mRNA in high vs low LG-ABN offspring ↑ CBP associated with exon 1_7_ GR promoter in high vs low LG-ABN offspring	High maternal care was associated with ↑ exp of GR mRNA and protein and ↑ binding of NGFI-A in the hippocampus. This correlated with ↓ exon 1_7_ GR promoter methylation and ↑Histone H3K9 acetylation Changes were persistent from early life to adulthood	[20, 43]
	Determine strain-specific epigenetic alterations of MS in mice	C57BL/6 J and DBA/2 J mice Hippocampus	MS: PND9, 24 h separation Assessed: 11–12 weeks	↔ Nr3c1 methylation in C57BL/6J mice ↑Nr3c1 CpG 13, 14 and 17 methylation in MS-treated DBA/2J mice	MS in DBA/2J mice ↑ methylation of CpG 13, 14 and 17 in Nr3c1 at 3 months of age	[45]
	Determine the effect of MS on exon 1_7_ GR promoter methylation	Sprague Dawley rats Hippocampus	MS: PND2–14, 3 h/day Assessed: PND21	↔ methylation of exon 1_7_ GR promoter or NGFI-A binding site ↑ NGF mRNA exp ↔NT-3	No effect of MS on methylation status of exon 17 of the GR promoter or the NGFI-A binding site in hippocampus	[44]
GR gene locus (7 million base pairs)	Determine the effect maternal care on DNA methylation and H3K9 acetylation of a region of Chr 18 containing the *Nr3c1* gene	Long-Evans Rats Hippocampus	High vs low LG- ABN Assessed: PND90	723 RDme and 471 RDac in GR gene were identified across the entire locus in high vs low LG-ABN Clustering patterns revealed changes in exp of *Pcdh* family genes. 20 out of 33 had ↑ exp in high compared to low LG-ABN offspring	Identified that variations in maternal care affect a broad genomic region and epigenetic and exp changes act on a family of genes localised in that broad genomic region Finding of the association of the *Pcdh* cluster of genes involved in synaptic plasticity	[46]
*Crh* promoter	Determine the effect of MS on *Crh* promoter methylation	Sprague Dawley rats Hypothalamus	MS: PND2–13, 4 h/day Assessed: PND61	↓ methylation of *Crh* promoter in hypothalamus (Met-C2) ↓ methylation of Met-C2 ↑ phosphoCREB binding to CRH CRE ↑ *Crh* hnRNA exp in hypothalamus (↔ amygdala) ↔ *Crh* mRNA exp in hypothalamus and amygdala	MS ↓ methylation of Met-C2 and ↑ transcriptional activity of *Crh* in the PVN on PND61	[47]
	Determine the effect of MS on *Crh* promoter methylation	Sprague Dawley rats Hippocampus CA1	MS: PND1–10, 3 h/day Assessed: 10 weeks of age	↑ H3 acetylation of the *Crh* promoter in MS vs no MS pups ↓methylation and ↓ binding of MeCP2 in the *Crh* promoter ↑*Crh* mRNA in MS vs no MS pups Enriched environment reversed the epigenetic up-regulation of *Crh*	MS ↑ acetylation of the *Crh* promoter region thereby allowing for increased transcriptional activity of *Crh* which was reversed when mice were treated to enriched environment	[48]
*Crfr2*	Determine the effect of MS on *Crfr2* methylation	C57Bl/6 mice Genomic DNA from sperm in F1 and F2 males Cortex in F2 females	Unpredictable MS: PND1–14, 3 h/day in F1 Assessed: 3–8 months of age	F1 ↓ methylation of 5′ CpG of *Crfr2* in M F2^a^ ↓ methylation of 5′ CpG of *Crfr2* in F Changes in gene exp were accompanied by ↓ mRNA exp	Early stress ↓ methylation of *Crfr2* and mRNA exp in adult C57/BL6 mice and that is transmitted across generations	[21]
*Avp* enhancer	Determine the effect of MS on *Avp* enhancer methylation and mRNA exp	C57Bl/6 mice PVN of hypothalamus	MS: PND1–10, 3 h/days Assessed: PND10, 6 weeks, 3 months and 1 year	↓ *Avp* enhancer methylation at 6 weeks, 3 months and 1 year ↓ methylation of CpGs largely mapped to CG13 of *Avp* enhancer from 6 weeks (↔ at PND10) ↓ binding of CG13 *Avp* enhancer at PND10 and 6 weeks ↓ methylation of CpGs w age (11 CpGs at 6 weeks, 7 at 3 months, 3 at 1 year) ↑ AVP mRNA exp from 6 weeks	MS causes ↓ methylation of the *Avp* enhancer from 6 weeks of age in C57/BL6 mice which is accompanied by persistent up-regulation of *Avp* exp in parvocellular neurons in the PVN	[23]
	Determine strain-specific epigenetic alterations of MS in mice	C57BL/6J and DBA/2J mice Hippocampus	MS: PND9, 24-h separation Assessed: 11–12 weeks	↑ methylation of CpG1 of *Avp* in MS treated M of C57BL/6J and DBA/2J strain	MS ↑ methylation of CpG 1 unit in the *Avp* enhancer DNA sequence	[45]

↑ increased, ↓ decreased, ↔ no change, *Avp* arginine vasopressin, *CBP* CREB binding protein, *Crfr2* corticotrophin-releasing hormone receptor 2, *Crh* corticotrophin-releasing hormone, *exp* expression, *F* female, *GR* glucocorticoid receptor, *h* hour, *hnRNA* heterogeneous nuclear ribonucleic acid, *LG-ABN* licking grooming arched-back nursing, *M* male, *mRNA* messenger ribonucleic acid, *MS* maternal separation, *NGF* nerve growth factor, *NGFI-A* nerve growth factor inducible protein A, *NT3* neurotophin 3, *PND* postnatal day, *PVN* paraventricular nucleus, *RDac* regional differences in acetylation, *RDme* regional differences in methylation, *Pcdh*: Protocadherin, *CA-1* Cornu Ammonis area 1, *MeCP2* Methyl CpG binding protein 2

^a^F2: MS M and control F were bred to produce F2 offspring

#### Glucocorticoid receptor gene

Pioneering studies on epigenetic alterations in GR promoter in response to variations in maternal care were first shown by Weaver and colleagues [20]. They reported increased methylation of the 5′ exon1_7_ GR promoter and decreased H3K9 acetylation both associated with reduction in GR messenger RNA (mRNA) expression in the hippocampus of pups raised by low licking grooming arched-back nursing (LG-ABN) dams [20]. Extended studies showed that increased 5′ cytosine phosphate guanine (CpG) site methylation in the low LG-ABN pups reduced binding of transcription factor nerve growth factor inducible protein A (NGFI-A) to GR exon 1_7_ promoter and reduced recruitment of CREB binding protein (CBP), subsequently reducing the levels of GR mRNA in hippocampus [20, 43]. These changes were observed both at postnatal day (PND) 6 (early) and PND90 (adulthood) suggesting the long-lasting nature of the epigenetic mark. In contrast, Daniels and colleagues reported no differences in the methylation status of exon 1_7_ GR promoter in maternally separated (MS) compared to control rats on PND21 [44]. The conflicting results could be due to differences in the early stress model (maternal care vs MS) and strain (Long-Evans vs Sprague Dawley) which may exert different effects on the epigenetic signature of the glucocorticoid receptor. Other studies also report epigenetic alterations in the GR promoter using different ELS models of rat/mouse strains [45, 46] (Table 2).

#### *Crh*

Chen and colleagues reported hypomethylation of the *Crh* promoter in the PVN of maternally deprived (MD) Sprague Dawley rats on PND61 [47]. This was associated with increased phosphoCREB binding to the *Crh* cAMP response element (CRE), critical in the regulation of transcription of *Crh*. Similarly, Wang and colleagues reported increased H3 acetylation and hypomethylation of the *Crh* promoter region in the hippocampal cornu ammonis 1 (CA1) region of rats with postnatal MS [48]. Franklin and colleagues reported hypomethylation of the CRH receptor 2 (*Crhr2*) in maternally separated male C57/BL6 mice at 3–8 months of age and demonstrated a transgenerational effect [21]. The hypomethylation was associated with decreased *Crhr2* gene expression which in this case was assessed in vitro using zebularine, a DNA methylation inhibitor [21]. This is in contrast to conventional understanding that DNA methylation is repressive [49, 50]; however, results correlate with the expected ELS-induced changes in HPA axis regulation and decreased *Crhr2* expression.

#### *Avp*

Murgatroyd and colleagues reported hypomethylation of the AVP enhancer sequence in the parvocellular neurons of the PVN [23]. Hypomethylation of the AVP enhancer was associated with increased *Avp* expression following ELS (maternal separation) from 6 weeks of age and was still evident at 1 year suggesting the long-lasting nature of this epigenetic mark. Mechanistic analysis using mouse hypothalamic-like cells revealed that the hypomethylated CpG sites bound MeCP2 during postnatal life that in turn recruited DNA methyl transferase (DNMTs) and histone deacetylase (HDACs) to regulate expression of *Avp* [23] and that MeCP2 occupancy further depended on polycomb binding earlier in hypothalamic development [51]. Contrasting results were reported in a more recent study showing hypermethylation of the *Avp* enhancer CpG site in the hippocampus of maternally deprived C57BL/6 and DBA/2 males [45]. This could be due to the type of tissue analysed and the fact that the CpG sites analysed by these two studies did not overlap.

### ELS-induced epigenetic changes in genes outside of the HPA axis

#### Brain-derived neurotrophic factor

ELS has been shown to decrease *Bdnf* expression whilst enhancing anxiety and depression-like behaviours [41, 52]. Roth and colleagues exposed Long-Evans rat pups to an abusive mother during the first week of life [53]. They reported hypermethylation of the *Bdnf* exons IV and IX in the prefrontal cortex on PND8 with associated reduction of *Bdnf* mRNA which persisted into adulthood. Bai and colleagues reported significantly decreased *Bdnf* mRNA and protein and increased miR-16 expression in the hippocampus of MD rats compared to those exposed to chronic unpredictable stress in adulthood (CUPS) and control rats [54] (see Table 3). The results suggested significant association of depression induced by MD to *Bdnf* and miR-16 levels but not the late-life stressors such as the CUPS thus emphasising the role of ELS-induced epigenetic alterations.

**Table 3:** Early stress-induced epigenetic modifications in other candidate genes

Candidate gene	Objective	Model/tissue type	Early stress/assessment age	Epigenetic and expression changes	Interpretation	References
*Er-α*	Determine the effect of maternal care on *Er-α* mRNA exp and *Erα1b* promoter methylation	Long-Evans hooded rats MPOA of Amygdala	Maternal care variations: high vs low LG-ABN Assessed: PND6	↓ *Erα1b* promoter methylation in high vs low LG-ABN offspring ↑ *Stat5* binding to *Erα1b* promoter in high vs low LG-ABN offspring ↑ *Er-α* mRNA exp in high vs low LG-ABN offspring	Higher levels of maternal care cause ↓*Erα1b* promoter methylation, ↑*Er-α* mRNA exp and ↑ Stat5 binding to *Erα1b* promoter in the MPOA in Long-Evans hooded rats on PND6	[59]
*5-HTT*	Determine the relationship between early stress and *5-HTT* gene methylation	Rhesus macaques Peripheral blood DNA	Rearing variations: MR vs NR Assessed: 90–120 days (infants)	↑ average *5-HTT* gene methylation in carrier of the low-expressing *rh5-HTTLPR* ↓ *5-HTT* mRNA in PBMCs	Variations in maternal care were associated with average differences in *5-HTT* gene methylation and this was dependent on the *5-HTT* polymorphism in Rhesus macaques	[57]
	Determine the effect of early stress on 5-HTT gene and whole genome DNA methylation	F bonnet macaques Peripheral blood DNA	VFD from 3–8 months age Assessed: mean = 8.4 years	↔ *5-HTT* and whole genome methylation ↑ *5-HTT* and whole genome methylation ↑ stress reactivity in VFD	*5-HTT* gene and whole genome methylation confer an ↑ stress reactivity following early stress in adolescent F bonnet macaques	[58]
*Gad1*	Determine the effect of maternal care on the *Gad1* promoter in the hippocampus	Long-Evans hooded rats Hippocampus	Maternal care variations: high vs low LG-ABN Assessed: 3–4 months of age except for NGFI-A association to *Gad1* promoter at PND4	↓ methylation of *Gad1* promoter in high vs low LG-ABN offspring ↑ histone H3K9 acetylation of *Gad1* promoter in high vs low LG-ABN offspring ↑ NGFI-A association w *Gad1* promoter in high vs low LG-ABN offspring ↑ *Gad1* mRNA exp in high vs low LG-ABN offspring	High levels of maternal care ↓ *Gad1* promoter methylation, ↑ *Gad1* promoter histone acetylation and ↑ *Gad1* mRNA in the hippocampus of Long-Evans hooded rats	[66]
*Bdnf*	Determine the effect of early stress on DNA methylation	Long-Evans hooded rats Hippocampus and PFC	Exposure to abusive or caring mother from PND1–7 for 30 min Assessed: PND8, 30 and 90	↑ methylation of *Bdnf* exon IV and IX in PFC ↓ *Bdnf* mRNA exp in PFC ↑ *Bdnf* mRNA exp in hippocampus	Early abuse ↑ methylation of *Bdnf* exon IV and IX in the PFC that persists into adulthood in Long-Evans hooded rats	[53]
	Determine the effect of early stress on *Bdnf* mRNA, protein and miR-16 exp	Sprague Dawley rats Hippocampus	MS: PND1–13 CUPS: 10–13 weeks C: no stress Assessed: 13–14 weeks Only *Bdnf* transcribed from 5ʹ exon VI and 3ʹ common exon analysed	↓ *Bdnf* mRNA exp in MS vs CUPS and C ↓ *Bdnf* protein in MS vs CUPS and C ↑ miR-16 exp in MS vs CUPS and C ↓ *Bdnf* mRNA exp in MS correlated significantly with ↑ miR-16 exp	MS induced significantly different BDNF and miR-16 expression in rats.	[54]
					BDNF and miR-16 levels were inversely related in the presence of early stress	
Reelin (*Reln*)	Determine the effect of MS on *Reln* expression	Wistar rats Hippocampus	MS: PND2–15 for 3 h Assessed: PND22	↑ *Reln* gene methylation ↓ *Reln* mRNA exp	MS ↑ *Reln* gene methylation and ↓ *Reln* mRNA exp in Wistar rats on PND22	[67]

↑ increased, ↓ decreased, ↔ no change, *5-HTT* serotonin transporter, *PND* postnatal day, *Bdnf* brain-derived neurotrophic factor, *DNA* deoxyribonucleic acid, *Er-α* estrogen receptor alpha, *exp* expression, *F* female, *Gad1* glutamic acid decarboxylase 1, *LG-ABN* licking grooming arched-back nursing, *MPOA* medial preoptic area, *MR* mother reared, *NR* nursery reared, *mRNA* messenger ribonucleic acid, *PBMC* peripheral blood mononuclear cells, *VFD* variable foraging demand, *MS* maternal separation, *NGFI-A* nerve growth factor inducible protein A, *PFC* prefrontal cortex, *PND* postnatal day, *Stat5* signal transducer and activator of transcription 5, *mi-R* micro-RNA, *CUPS* chronic unpredictable stress, *C* control

#### Serotonin transporter (5-HTT or *Slc6a4*) gene

Reduction in *5-HTT* expression has been observed in response to ELS in non-human primate and rodent models [55, 56]. Kinnaly and colleagues investigated the role of ELS-induced epigenetic modifications in the *5-HTT* gene using non-human primate models [57, 58] (see Table 3). These studies assessed the relationship between *5-HTT* expression and *5-HTT* gene methylation following ELS in infant rhesus macaques [57] and adolescent bonnet macaques [58]. No significant effect of rearing was observed on the *5-HTT* methylation status in either study. However, carriers of the short allele of the *5-HTT* polymorphism had higher mean *5-HTT* CpG methylation, and this was significantly associated to the levels of peripheral *5-HTT* expression in the infant rhesus macaques [57]. In adolescent and adult Bonnet macaques, genome-wide methylation levels were associated with *5-HTT* expression in those exposed to early stress as infants compared to controls [58]. The above studies suggest that *5-HTT* gene methylation is not susceptible to variations in maternal care; however, polymorphisms in the *5-HTT* gene and methylation at other *cis* or *Trans* regulating sites as may be conferring increased stress reactivity.

#### Estrogen receptor-α gene

Increased expression of estrogen receptor-α (ERα) in response to variations in maternal care was first reported by Champagne and colleagues using the LG-ABN rat model [39]. The study reported elevated ERα mRNA expression in the medial preoptic area of high LG-ABN dams compared to low LG-ABN, and this effect was transmitted to the female offspring of the high LG-ABN dams [39, 59]. Champagne and colleagues then demonstrated decreased ERα1b promoter methylation in high vs low LG-ABN offspring on PND6 [59] (Table 3). It was characterised that this hypomethylation of the promoter region enhanced binding of the STAT5 transcription factor to the ERα1b promoter and a corresponding increase in ERα mRNA expression in response to increased maternal care.

#### Glutamate decarboxylase-1 and Reelin genes

Glutamate decarboxylase 1 (GAD1) is a key enzyme in the synthesis of gamma amino-butyric acid (GABA), and Reelin (*Reln*) is important in migration of new neurons in the central nervous system (CNS). Postmortem studies of schizophrenic patient brains have shown decreased forebrain expression of *GAD1* [60, 61] and increased methylation of the *GAD1* promoter [62, 63] and decreased *RELN* expression [64, 65]. Zhang and colleagues assessed the methylation status of the *Gad*1 promoter in the offspring of the high vs low LG-ABN dams [66] (See Table 3). Hypomethylation, increased histone H3K9 acetylation and increased NGFI-A association with the *Gad1* promoter were observed in those raised by high LG-ABN dams leading to increased *Gad1* mRNA expression [66]. Qin and colleagues reported hypermethylation of the *Reln* gene and subsequent down-regulation of Reln mRNA in the hippocampus of Wistar rats exposed to MS from PND2–15 compared to controls [67].

## Early stress-induced epigenetic modifications in humans

### Epigenetic alterations in candidate genes

In human studies, most focus to date has been on the role of GR due to its negative feedback control in stress responsivity and serotonin transporter (*5-HTT*) due to its polymorphisms and role in mediating ELS and later-life stress effects on adult depression status [14, 68] (see Table 4 for summary of studies).

**Table 4:** Early stress-induced epigenetic modifications of candidate genes in humans

Candidate gene	Objective	Model/tissue type	Early stress/assessment age	Epigenetic and expression changes	Interpretation	References
rRNA promoter	Determine the effect of childhood abuse on methylation status of the rRNA promoter	Retrospective Hippocampus	CA + suicide vs no CA + no suicide Assessed Mean age 35 yrs. *N* = 30	↑ methylation rRNA promoter and 5′ regulatory region in CA ↑ methylation of 21 of 26 CpG sites in CA ↓ rRNA expression in hippocampus	Childhood abuse ↑ methylation of rRNA promoter in the hippocampus of suicide completers in adulthood	[98]
GR promoter	Determine the effect of childhood abuse on the methylation status of the GR promoter and GR mRNA exp	Retrospective Hippocampus	Child abuse Assessed Mean age 34.6 yrs. *N* = 36 M	↑ methylation of CpGs in GR promoter ↓ GR mRNA exp ↓ GR 1F mRNA exp	Childhood abuse ↑ methylation of individual CpGs within the GR promoter and GR1F exon resulting in ↓ GR and GR 1F mRNA expression in adult hippocampus	[19]
	Determine the effect of early stress on methylation status of the GR promoter	Retrospective Peripheral blood WBCs	Early stress^a^ Assessed: mean age 27.4 yrs. *N* = 99	↑ CpG1 methylation w ↓ parental care or loss ↑ CpG3 methylation w maltreatment or parental loss ↔ CpG2, 4, 5–13 methylation w childhood adversity	Early stress ↑ methylation of individual CpGs of the GR promoter in adulthood	[71]
	Determine the effect of childhood maltreatment and severity on *NR3C*1 promoter methylation	Retrospective Peripheral blood WBCs	Childhood maltreatment^b^ Assessed: mean ages and sample size BPD 30.76 yrs. *N* = 101 MDD 41.63 yrs. *N* = 99 MDD + PTSD 37.33 yrs. *N* = 15	↑ CpG 2–8 methylation of the *NR3C1* gene promoter in maltreated participants ↑ severity of maltreatment ↑ methylation status of *NR3C1* except for CpG1 site	Childhood maltreatment was associated with ↑ methylation of the *NR3C1* gene promoter in peripheral blood in adulthood. Number and severity of maltreatment correlated positively with methylation status	[70]
*NR3C1* locus (6.5 million base pairs)	Determine the effect of CA on a 6.5 Mbp loci centred on *NR3C1*	Retrospective Hippocampus	CA + suicide vs no CA + no suicide Assessed: adulthood *N* = 24	281 DMRs were identified ↑ methylation in 126 DMRs in controls ↑methylation in 155 DMRs in abused 3 clusters of DMRs mapped within α-, β- and γ- protocadherin (*PCDH*) gene family DMRs enriched more in *α-PCDH* in the abused brains	Early stress has a broader epigenomics imprint expanding to promoters of genes both upstream and downstream to the *NR3C1* gene and includes the *PCDH* gene family implicated in synaptic plasticity	[69]
*5HTT* or *SLC6A4*	Determine the effect of CA on *SLC6A4* promoter methylation and mRNA expression	Retrospective Peripheral blood DNA	Childhood adversity^a^ Assessed: adulthood *N* = 102 MDD with or without CA	↑average methylation in promoter of *SLC6A4* with CpG7 showing higher methylation in CA to no CA ↑ methylation in CpG2 of *SLC6A4* associated with physical abuse	Childhood adversities were significantly associated with higher *SLC6A4* promoter methylation in people with current MDD	[76]
	Determine the effect of CA on the methylation status of *SLC6A4* promoter	Retrospective Lymphoblast cell lines	Childhood maltreatment Assessed: mean age: M 49 yrs. F 47 yrs. *N* = 192	↑ overall *SLC6A4* promoter methylation in abused male and female ↑ methylation of CpG1 and CpG 3 of *SLC6A4* in abused females compared to non-abused ↔ in individual CpG sites in males	Childhood abuse increased methylation of CpGs in the promoter of *SLC6A4* in adults	[74, 75]

↑ increased, ↓ decreased, ↔ no change, *yrs.* years, *CA* child abuse, *DNA* deoxyribonucleic acid, *mRNA* messenger ribonucleic acid, *exp* expression, *rRNA* ribosomal ribonucleic acid, *GR* glucocorticoid receptor, *M* male, *F* Female, *WBC* white blood cell, *BPD* borderline personality disorder, *PTSD* post-traumatic stress disorder, *MDD* major depressive disorder, *DMRs* differentially methylated regions, *PCDH* protocadherin, *NR3C1* nuclear receptor subfamily 3, group C, member 1, *SLC6A4* solute carrier family 6, member 4 (neurotransmitter transporter)

^a^Early stress included: low levels of parental care, parental loss and childhood maltreatment

^b^Sexual, physical and emotional abuse; BPD had higher childhood maltreatment; MDD had lower maltreatment

### GR gene

McGowan and colleagues were the first to demonstrate hypermethylation of the GR promoter exon 1_F_ and decreased GR mRNA in the hippocampus of adults who were exposed to childhood abuse [19]. When compared to suicide victims or controls with no childhood abuse the GR promoter of suicide victims with a history of childhood abuse showed a significant increase (*p* < 0.05; *d* = 1.07) in methylation. This study was thus able to translate the results previously described in LG-ABN rat model [43]. McGowan and colleagues extended research by analysing a wider locus containing the GR gene on chromosome 5 and reported differential methylation in promoters of the protocadherin (*PCDH*) gene family which are implicated in synaptic plasticity [69]. Whilst McGowan and colleagues analysed the epigenetic changes in hippocampal GR gene, other human studies have reported similar hypermethylation GR gene promoter in peripheral blood leukocytes of adults exposed to early childhood stress [70, 71]. Murgatroyd and colleagues have recently demonstrated maternal stroking to modify CpG methylation within this GR region further translating the rat LG results [72]. These studies have shown that hypermethylation of specific CpG sites were associated to type of early stress or the severity of early maltreatment (see Table 4).

### *5-HTT* gene

Philibert and colleagues were the first to report hypermethylation of the *5-HTT* promoter and subsequent reduction in *5-HTT* mRNA; however, this study did not assess the role of early maltreatment [73]. Further work from Beach and colleagues reported association of increased methylation of the *5-HTT* promoter to childhood abuse. In their 2010 study, Beach and colleagues report significant association of overall methylation of the 5-HTT promoter region (CpGs analysed = 71, CpGs used in analyses = 26) to childhood abuse (*p* < 0.0004, *d* = 0.73) [74]. In particular, increased methylation of the sites CpG1 and CpG3 in the 5-HTT promoter was significantly associated in abused females. In their 2011 study, the same group showed significant association of childhood abuse to 5-HTT promoter methylation, 5-HTT mRNA levels and a correlation to 5-HTT genotype and adult anti-social personality disorder [75]. The study reported that 9 % of the variation in the personality disorder was attributed to increased 5-HTT promoter methylation observed only in those that were sexually abused (see Table 4 for details). Kang and colleagues also reported similar hypermethylation of the *5-HTT* promoter (total of seven CpG sites) in abused compared to non-abused people with current MDD [76]. The average methylation percentage of the seven CpG sites for any adversity was significantly higher in those who were abused in childhood (*p* < 0.001, *d* = 1.1).

### Genome-wide methylation

A number of studies have assessed the effect of early stress on genome-wide methylation patterns (See Table 5 for summary of studies). Naumova and colleagues demonstrated hypermethylation of 28 genes involved in brain development and function including those regulating the arginine vasopressin 1A receptor (*AVPR1A*), GABA A receptor (*GABRA5*), glutamate receptor (*GRM5*), among others, in 7- to 10-year-old children following institutionalisation [77]. Hypermethylation of candidate genes has also been shown in adulthood, with Labonte and colleagues reporting hypermethylation of 248 gene promoters and associated decreases in mRNA expression of these promoters in the hippocampi of adult men who completed suicide [78]. Genes responsible for cellular and neuronal plasticity were the most differentially methylated, including the alsin (*ALS2*) gene promoter. Many other studies have also shown significant global methylation differences between those exposed to early adversity [79–81]. These studies suggest that alterations in the early environment have the ability to cause changes in the methylation status of numerous genes across the genome, including those involved in control of nervous and immune system development and function.

**Table 5:** Effects of early stress on genome-wide methylation in humans

Objective	Model/tissue type	Early stress/assessment age	Epigenetic and expression changes	Interpretation	References
Determine the effect of childhood SES on genome-wide methylation in adulthood	Retrospective Peripheral blood DNA	High vs low childhood SES Assessed: 45 yrs. *N* = 40 M	666 gene promoters ↑ and 586 promoters ↓ methylation in high vs low childhood SES The genes involved fall into extra and intracellular signalling, DNA signalling and metabolic signalling categories.	Variations in childhood SES cause changes in genome-wide methylation in adulthood with genes in extra and intra cellular signalling and metabolic functioning	[79]
Determine the effect of early environment on genome-wide methylation levels	Retrospective Peripheral blood DNA	Institutional care vs raised by biological parents Assessed: mean 8.25 yrs. *N* = 28	Differential methylation of 914 of 26,214 CpG sites from 838 gene promoters across groups^a^ ↑ methylation of 744 promoters in institutionalised ↓ methylation of 94 promoters in institutionalised Promoters mainly involved in control of cellular signalling and the immune response	Early environmental alterations cause changes in methylation of a number of genes important for control of cellular signalling and the immune response in childhood	[77]
Determine the effect of childhood abuse on genome-wide DNA methylation	Retrospective Hippocampus	CA + suicide vs no CA + suicide Assessed: adulthood *N* = 41 M	Differentially methylated promoters in CA vs no CA were spread across the genome 248 (68.5 %) promoters ↑ methylation in CA 114 (31.5 %) promoters ↓ methylation in CA ↑ methylation associated w ↓ mRNA exp	Childhood abuse causes alteration in the methylation of gene promoters and mRNA exp in adulthood specifically genes involved in neuronal plasticity	[93]
	Retrospective Peripheral blood DNA	PTSD + CA vs PTSD + no CA Assessed: mean age CA = 39.6 yrs. No CA = 43.69 *N* = 61	Differential methylation in promoters of abused vs non-abused PTSD patients ↑ methylation in transcripts of PTSD + CA group (11.78 %) vs PTSD + no CA (0.8 %) 14 transcripts differentially methylated in CA vs no CA	Childhood abuse causes alteration to the methylation of CpG sites in both promoter regions and gene body and include specifically genes involved in CNS development in the abused PTSD group	[80]
Determine the effect of childhood abuse in methylation status of immune system and cytokine regulation	Retrospective Peripheral blood DNA	PTSD + CA, PTSD + no CA, C + CA, C + no CA Assessed: adulthood *N* = 110	↑ global methylation in PTSD vs C ↔ methylation due to CA in PTSD or C Gene specific associations with found in *BDNF*, *HSF1*, *TLR8* for PTSD and CA ↑plasma TNFα in CA vs no CA	Childhood abuse early in life can alter global and gene specific DNA methylation patterns specifically involved in immune dysregulation	[81]

↑ increased, ↓ decreased, ↔ no change, *yrs* years, *SES* socioeconomic status, *M* Male, *DNA* deoxyribonucleic acid, *mRNA* messenger ribonucleic acid, *exp* expression, *CA* childhood abuse, *PTSD* post-traumatic stress disorder, *C* controls, *BDNF* brain-derived neurotrophic factor, *HSF1* heat shock transcription factor 1, *TLR8* toll-like receptor 8, *TNFα* tumour necrosis factor alpha, *CNS* central nervous system

^a^Genes modified by rearing environment include those involved in control of the dopaminergic system (*TERF2IP*), serotonin biosynthesis and serotonin receptor activity (*TPH*, *HTR1D*, *HTR1F*), glucocorticoid and steroid biosynthesis and their receptor activity (*NRIP1*, *PPARGC1B*, *UGT*), genes encoding the arginine vasopressin receptor, glutamate, cadherin and cholinergic receptors, and others which are collectively responsible for neural communication, memory formation and learning and retention

## Discussion

As evidenced in the studies reviewed above, changes in DNA methylation in response to the early environment remain the best characterised to date. This could be due to DNA methylation being a robust epigenetic mark and available to study across lifespan and generations [21, 82]. More recent studies however have also analysed the role of microRNAs [54], histone modifications [83] and DNA hydroxymethylation [84] in mediating early stress effects.

Results from the animal models reviewed suggest that ELS exerts significant methylation changes in the stress-regulatory genes. Enhanced *Crh* and *Avp* expression in the hypothalamus may lead to a hypersensitive HPA axis [23, 47], whilst reduced *Crhr2* and GR expression (in the hypothalamus and hippocampus respectively) may affect negative feedback of the HPA response to stress [20, 21]. Epigenetic signatures in the GR gene promoter in humans exposed to a variety of early adversity translate the results observed in animal models and confirm the central role of GR methylation in the dysfunctional negative feedback of HPA axis [19, 70, 71]. Analysis of a broader GR locus (~7 Mpb centred with *Nr3c1*) in both rats and humans exposed to early adversity revealed conserved methylated sequences in the GR locus including the GR promoter methylations thus emphasising an evolutionary role of the GR locus in stress responsivity [46, 69] (See Tables 2 and 4 for details).

Studies of animal models have centred on other candidate genes implicated in the pathogenesis of neuropsychiatric disorders. Altered 5-HTT expression early in life may moderate the impact of early environment on emotion development [28] as also shown by pharmacological blocking studies [85, 29]. The findings on *5-HTT* promoter methylation therefore support the role of reduced 5-HTT activity in the onset of adult depression-like behaviours [57, 58]. Reduced *BDNF* expression has been implicated in anxiety and depression-like behaviours [41, 52]; therefore, hypermethylation of the *Bdnf* gene alongside reduced *Bdnf* mRNA expression in the prefrontal cortex (PFC) of rats exposed to abuse (67) suggests early stress may play a role in the development of anxiety and depression disorders. In humans exposed to early adversity, *5-HTT* hypermethylation has been associated to adult depression status depending on the polymorphic status of the *5-HTT* gene, emphasising the role of genetic polymorphisms on epigenetic effects [14, 76]. Interestingly, studies to date have only analysed the effect of prenatal stress on the methylation status of the *BDNF* gene in humans [86, 87] and not variations in postnatal stress. BDNF is known to regulate neuronal development, serotonergic functions and signalling [88]. For example, BDNF promotes the development and function of serotonergic neurons where high affinity receptors for BDNF, Trkb, are also found [89]. Therefore, ELS-induced reduced BDNF could also potentially lead to decreased function of the serotonergic system thus leading to mood and affective behavioural dysfunctions as reported in the above studies. In addition, BDNF has been shown to be regulated by estrogen via ERα within the hippocampus of rat brains [90]. In this context, the increased ERα expression in the brains of the high LG-ABN rats [59] suggests that estrogen is important in regulating normal brain development and possibly involves maturation of neural systems via BDNF regulation. The down-regulation of Reelin post ELS [67] may negatively impact hippocampal function, potentially altering stress axis regulation and predisposing to anxiety and depression across the lifespan. A neurodevelopmental origin for schizophrenia is shown from the study suggesting increased methylation and reduced *Gad1* expression in rats [66] which fits with the observations in postmortem brains of schizophrenic patients [62, 63].

One of the major questions in psychiatric epigenetics is whether epigenetic changes observed in the periphery reflect the changes in the brain. For example, hypermethylation of GR gene promoter and subsequent increase in GR mRNA was reported in both peripheral leukocytes [70, 71] and in the neurons of the hippocampus [19]. Analysis of methylome of cells in the PFC and T cells in mother vs surrogate-reared rhesus macaques revealed similarities in the methylation of cells in PFC and T cells, specifically the GR receptor promoter region alongside genes in the immune response, transcription and response to stimulus [91]. These findings suggest that peripheral GR methylation or cell specific (example T cells) methylation changes could be a potential biomarker for assessing early stress-induced HPA dysfunction. Genome-wide methylation studies show that childhood maltreatment leaves a systemic, genome-wide mark by altering the methylation status of key genes in regulatory pathways such as intra and extra cellular signalling [77, 79, 80, 92, 93]. Although most work to date has focused on the stress response, it may be interesting to consider whether prolonged immune dysfunction that often persists after early stress is secondary to epigenetic changes in immunoregulatory genes as reported by Smith and colleagues in PTSD [81] or if it results indirectly from epigenetic changes in stress-regulatory genes.

## Conclusions

Research to date highlights the remarkable susceptibility of the genome, and particularly of genes involved in stress and emotion regulation, to environmental alterations early in the lifespan. Many of these changes persisted into adulthood, and epigenetic mechanisms have been shown to contribute to the long-lasting nature of ELS and its contribution to an individual’s disease risk and susceptibility to neuropsychiatric conditions across the lifespan.

## Abbreviations

5-HTT: 5-hyrodxytryptamine transporter (serotonin transporter)
ACTH: adrenocorticotrophic hormone
AVP: arginine vasopressin
BDNF: brain-derived neurotrophic factor
CA1: cornu ammonis 1
CpG: cytosine phosphate guanine
CRH: corticotrophin-releasing hormone
DNMT: DNA methyl transferase
ELS: early life stress
GAD1: glutamate decarboxylase 1
GR: glucocorticoid receptor
HDAC: histone deacetylase
HPA: hypothalamic-pituitary-adrenal axis
MD: maternal deprivation
MS: maternal separation
PCDH: protocadherin
PFC: prefrontal cortex
PND: postnatal day
PVN: para ventricular nucleus
